# A 3D Model of Human Trabecular Meshwork for the Research Study of Glaucoma

**DOI:** 10.3389/fneur.2020.591776

**Published:** 2020-12-01

**Authors:** Sara Tirendi, Sergio Claudio Saccà, Stefania Vernazza, Carlo Traverso, Anna Maria Bassi, Alberto Izzotti

**Affiliations:** ^1^Department of Experimental Medicine (DIMES), University of Genoa, Genoa, Italy; ^2^Inter-University Center for the Promotion of the 3Rs Principles in Teaching & Research (Centro 3R), Pisa, Italy; ^3^Ophthalmology Unit, Istituto di Ricovero e Cura a Carattere Scientifico Ospedale Policlinico San Martino, Genoa, Italy; ^4^Istituto di Ricovero e Cura a Carattere Scientifico, Fondazione Bietti, Rome, Italy; ^5^Clinica Oculistica, Dipartimento di Neuroscienze, Riabilitazione, Oftalmologia, Genetica e Scienze Materno Infantili, University of Genoa and Istituto di Ricovero e Cura a Carattere Scientifico Ospedale Policlinico San Martino, Genoa, Italy; ^6^Mutagenesis Unit, IST National Institute for Cancer Research, Istituto di Ricovero e Cura a Carattere Scientifico San Martino University Hospital, Genoa, Italy; ^7^Department of Health Sciences, University of Genoa, Genoa, Italy

**Keywords:** glaucoma pathogenesis, trabecular meshwork, endothelial dysfunction, extracellular matrix, aqueous humor proteome

## Abstract

Glaucoma is a multifactorial syndrome in which the development of pro-apoptotic signals are the causes for retinal ganglion cell (RGC) loss. Most of the research progress in the glaucoma field have been based on experimentally inducible glaucoma animal models, which provided results about RGC loss after either the crash of the optic nerve or IOP elevation. In addition, there are genetically modified mouse models (DBA/2J), which make the study of hereditary forms of glaucoma possible. However, these approaches have not been able to identify all the molecular mechanisms characterizing glaucoma, possibly due to the disadvantages and limits related to the use of animals. In fact, the results obtained with small animals (i.e., rodents), which are the most commonly used, are often not aligned with human conditions due to their low degree of similarity with the human eye anatomy. Although the results obtained from non-human primates are in line with human conditions, they are little used for the study of glaucoma and its outcomes at cellular level due to their costs and their poor ease of handling. In this regard, according to at least two of the 3Rs principles, there is a need for reliable human-based *in vitro* models to better clarify the mechanisms involved in disease progression, and possibly to broaden the scope of the results so far obtained with animal models. The proper selection of an *in vitro* model with a “closer to *in vivo*” microenvironment and structure, for instance, allows for the identification of the biomarkers involved in the early stages of glaucoma and contributes to the development of new therapeutic approaches. This review summarizes the most recent findings in the glaucoma field through the use of human two- and three-dimensional cultures. In particular, it focuses on the role of the scaffold and the use of bioreactors in preserving the physiological relevance of *in vivo* conditions of the human trabecular meshwork cells in three-dimensional cultures. Moreover, data from these studies also highlight the pivotal role of oxidative stress in promoting the production of trabecular meshwork-derived pro-apoptotic signals, which are one of the first marks of trabecular meshwork damage. The resulting loss of barrier function, increase of intraocular pressure, as well the promotion of neuroinflammation and neurodegeneration are listed as the main features of glaucoma. Therefore, a better understanding of the first molecular events, which trigger the glaucoma cascade, allows the identification of new targets for an early neuroprotective therapeutic approach.

## Introduction

Primary open angle glaucoma (POAG) is a chronic disease that leads to retinal ganglion cell (RGC) loss and, consequently, the characteristic cupping of the papilla at the optic nerve level. In spite of the fact that it has been known since the time of Hippocrates, many aspects of this disease still remain obscure.

The only therapy recognized to be useful for glaucoma treatment is the lowering of intraocular pressure (IOP) although the exact relationships between IOP elevation and the optic nerve head (ONH) degeneration, which leads to visual field alteration, have not been understood yet. Indeed, IOP reduction alone is not always enough for slowing-down blindness progression (1, 2).

Many molecular mechanisms such as the ones involved in glaucoma etiology have been recognized, and in this regard, a wide range of substances with action either on a specific target or on multiple targets have been discovered (3).

Neuroprotection, neurodegeneration, and neuroenhancement have gained great importance over time because such approaches not only prevent RGCs from death but also repair or regenerate the cell damage, changing the course of the disease (4).

The primary molecular damage that occurs, which may be either of a degenerative/ischemic or a mechanical/metabolic nature, is able to induce changes in the extracellular matrix (ECM) of trabecular meshwork (TM) and in the TM itself that lead to retinal ganglion cell degeneration (5–8). The neuroprotective approach helps prevent these outcomes by improving neural recovery after pathologic insult, which results in a neural system optimization.

In glaucoma, the RGC degeneration induces trans-synaptic alterations capable of involving the entire visual chain up to the calcarine fissure (9). In these regard, any compounds able to interfere with the cascade of event that leads to visual field degeneration can be considered neuroprotective.

A wide variety of experimentally inducible animal glaucoma models ranging from large animals (i.e., non-human primates, cattle, dogs, and cats) to small animals (i.e., rats, mice, and zebrafish) are used in glaucoma research. Each of these animal models can help explain some molecular aspects of such a complex disease. Generally, however, the use of large animals offers better access to eye structures compared to the use of small ones due to larger eye size. In addition, non-human primates also show a resemblance to human ocular anatomy. However, for ethical and economic reasons, small animals such as rodents have gained ground in the research community. Among rodent models, the DBA/2J mouse is widely used because the mutations (*Tyrp*1^b^ and *Gpnmb*^R150X^) it presents cause a pigment dispersion syndrome similar to that found in humans and allows for the study of the effects of elevated IOP on the retina and optic nerve head (10–12).

There are several experimental manipulations to induce glaucoma in animal models that aim either to direct damage to ganglion cell axons (13–15) or indirect through IOP elevation (16, 17). In addition, mice can be genetically manipulated in order to express mutated human genes such as the MYOC Tyr437His mutation, which is responsible for an autosomal dominant form of human juvenile glaucoma (18).

Unfortunately, due to the heterogeneity of the disease and the addition of comorbidities, it is difficult to find an animal capable of reproducing the entire disease (19, 20). Moreover, the most common experimental techniques used for inducing IOP elevation [i.e., laser photocoagulation of entire trabecular meshwork (TM), intracameral injection either of latex microspheres or autologous fixed red blood cells to blockade TM, topical application of prednisolone, and so on] (21–24) cause an irreversible damage to the TM, which is the main tissue involved in the onset of the high-tension glaucoma cascade.

In humans, progressive TM degeneration is considered the “primum movens” for the decrease in outflow facility and, consequently, for the increase in IOP hypertension. Therefore, if in most animal models the TM is destroyed, these models may not provide complete information on glaucoma development (25, 26). Furthermore, the use of young animals as glaucoma models may represent an oversimplification of glaucoma issues because they do not show all those age-related factors that in human conditions promote and characterize glaucoma, i.e., genomic, biochemical, cellular, and system biology alterations (27–29). However, few studies on middle-aged or elderly animal models have not shown reliable results (30).

The proper measures to use animals of different ages as experimental models include a careful use of anesthetic agents as well as the attention for both physical decay and stress conditions (31).

However, differences in response to dexamethasone treatment were observed between young and elderly rabbits. Indeed, only the younger rabbits showed a hypertensive response probably due to their immature irido-corneal angle (32–34).

From a molecular point of view, glaucoma can be defined as a syndrome in which proapoptotic signals toward the head of the optic nerve (ONH) lead to glaucoma typical morpho-functional alterations such as both anterograde and retrograde RGC degeneration and trans-synaptic anterograde degeneration (35).

Given the fact that in high-tension glaucoma the TM plays a fundamental role in its pathogenesis, several previous studies (26, 36–38) have hypothesized that either the functional decay of TM cells or the TM cellularity depletion lead to ocular hypertonus, which produces deleterious effects for both the outflow and the RGCs.

TM is essential for the passage of the aqueous humor through the conventional outflow pathway. Indeed, after damage, TM cells change their gene expression (25) encoding for proteins which, from the anterior to the posterior segment, become pro-apoptotic signals for the ganglion cells and the retina.

For the past 20 years, in addition to animal models, also animal- or human-derived *ex vivo* and *in vitro* models have been used for glaucoma study. In particular, these studies have the aim to fill the gap left by animal models. The main studies are focused on the role of oxidative stress in promoting cell/tissue defects found in glaucomatous patients (39). Over time, the continuous progress in the field of cell cultures has been able to improve the cell environment and to recreate a condition “closer to *in vivo*” (i.e., biocompatible scaffolds, bioreactors, lab-on-a chip) (40, 41). Moreover, *in vitro* models compared to *ex vivo* ones have the advantage of overcoming the problem of a limited incubation period, making longer experimental times possible (42).

The purpose of this review is to summarize our team's research progress using a 3D advanced human model of TM, arguing that the 2D model has limitations and hoping that our work may improve the performance in glaucoma studies.

## 3D Cell Cultures As A Research Model

Both animal and *in vitro* cell culture models are widely used in research to improve the knowledge about the mechanisms of disease onset and propagation and the development of preclinical drugs. However, the inconsistencies of animal researches and the oversimplification of conventional two-dimensional *in vitro* models have frequently delayed therapeutic advances, like in the case of glaucoma. Indeed, not all promising discoveries and treatments obtained from such models have given favorable outcomes with human evidence. Among the reasons behind animal model limitations, the poor standardization of experimental procedures and the variation of environmental conditions as well as interspecies variation (e.g., genetic differences) between animals are listed as the most relevant (43). In addition, in two-dimensional (2D) culture models, the loss of specific tissue function and physiology leads to a lack of predictability in terms of physiological significance and clinical response prediction (44).

One of the most important developments in this field has been the use of micro-engineering techniques for culturing cells in a three-dimensional (3D) system. Anyway, a more sophisticated *in vitro* model does not need to recapitulate every aspect of an animal model or human responses, but it needs to provide predictive data for a particular question (45).

Three-dimensional culture models aim to mimic the proper interactions of both cell–cell and cell–environment providing for the complex biochemical and physical signals as found in *in vivo* tissue structure (46, 47). Indeed, the maintenance both of cellular morphology and polarity enables gene expression, signaling, and metabolism similar to source tissue (48–50).

In order to accomplish this, the study of specific cell types (i.e., osteoblasts, hepatocytes, lymphocytes, trabecular meshwork cells, and so on) provides for an up-stream analysis both of structural architecture of tissue-derived cells and of the matrices/scaffold composition in which to embed the various cell types to obtain the proper 3D microenvironment (44, 47, 51).

In 3D cultures, matrices/scaffolds from several materials, within their structure, support cell growth, organization, and differentiation. Indeed, either biomaterials (e.g., natural or synthetic hydrogels) (52, 53) or the fabrication processes (e.g., electro-spinning, particulate-leaching, and solid free-form fabrication techniques) (54–56) used for these matrices/scaffolds confer mechanical and physical properties to them as well as other features including porosity and permeability, which provide the architecture for cellular supports.

Ideally, 3D cell culture matrices are able to recreate the extracellular cellular matrix (ECM) features to better mimic *in vivo* environments. As is known, the interaction between cells and ECM provide all those biophysical and biochemical functions including the transport of soluble signaling molecules, nutrients, and waste metabolites as well as mechanical integrity; to a certain extent, therefore, these matrices have to reflect the features of specific tissue ECM to each application (57–59). Mechanical properties of 3D matrix are important also because they can directly drive cell traction forces influencing both shape and responses of the cells. For example, the flattened shape of 2D cultures is due to the stiffness of the surfaces of the support to which they adhere (e.g., micro-well plates, tissue culture flasks, and Petri dishes) (60).

As mentioned above, among the biomaterials for cell embedding, polymers that form natural or synthetic hydrogels are mainly used. When mixed with cells, these polymers undergo fast and gentle polymerization, after which they can be degraded hydrolytically or enzymatically. However, natural hydrogels such as Matrigel^TM^, fibrin gel, and alginate, compared to synthetic ones (e.g., PEG, peptide, and DNA gels) are more used both because of their better biocompatibility and mild gelification (47, 61).

## 3D *In Vitro* Glaucoma Models

The advancement in *in vitro* model approaches for the study of glaucoma allowed the investigation of cellular behavior at molecular level in order to ameliorate the knowledge of disease onset and progression. Although *in vitro* models are not representative of the intricacy of glaucomatous disease, they have provided significant results when used for cell cultures, tissue cultures or *ex vivo* preparations. Additionally, according to the 3Rs principles, the use of the so-called alternative methods of which *in vitro* approaches are part promote the reduction in number of animals used in experimental tests and the replacement of animals when it is possible (62).

In this regard, several studies have mostly focused on setting up 3D models of TM, given its crucial role in conventional outflow pathway and in high-tension glaucoma onset.

Primary human trabecular meshwork (HTM) cells, for instance, were isolated from both juxtacanalicular and corneoscleral region, after which multiple HTM cell layers were included into a highly porous membrane, SU-8 scaffold, with predefined and well-controlled microstructure dimensions by standard photolithography techniques (63, 64). The bioengineered 3D HTM cell structures mimicked the TM structure found in *in vivo* conditions in terms of their spatial distribution, ECM synthesis/secretion, as well as their responsiveness after elevated hydrostatic pressure (EHP)-induced mechanical strain and drugs, i.e., latrunculin-B or steroids.

In a study on primary porcine TM cells, high viability and proliferation after seeding into a highly porous matrix of natural biopolymer construct, the collagen-chondroitin sulfate scaffold, was reported (65).

Moreover, *in vitro* models of 3D HTM cells (66) and 3D multipotent progenitors from HTM (67) were also obtained by embedment in a natural hydrogel scaffold such as Matrigel^TM^. In this regard, the 3D HTM cells obtained in this way have shown, besides morphological and architectural changes after dexamethasone, TGFβ2 and benzalkonium chloride (BAK) stimulations, the upregulation of proinflammatory cytokines and MMPs after BAK treatment, and their migration through basement membranes. Therefore, the use of degradable scaffolds, compared to permanent ones, also offers an opportunity for further molecular investigations.

The second Matrigel^TM^-based study revealed that the embedment of TM stem cell populations did not allow for cell expansion but provided the normal phenotype restoration able to express not only ESC and NC markers but also the TM ones (68).

However, all these 3D models of TM from natural or synthetic scaffolds have recapitulated important features of *in vivo* TM tissue morphology, highlighting the huge progress in tissue engineering in faithfully mimicking the native tissue.

In addition to innovative 3D TM cultures, a new *in vitro* approach based on lab-on-a-chip (LOC) technology has very recently been proposed, which consisted of a high-controlled EHP system and microculture system of purified primary rat RGCs (41). Such system has proved a useful tool to investigate the neuroprotective role of growth factors and mimic peptide in preserving the RGC death after EHP.

Over the years, in order to mimic the effects of oxidative stress in glaucoma *in vitro* models, repeated exposures of TM cells to different H_2_O_2_ concentrations (ranking from 100 μM to 1 mM) were evaluated (6, 69–72). H_2_O_2_ is the most widely used pro-oxidant because it easily crosses the cell membranes and, in presence of iron ions, produces reactive hydroxyl radicals, which are considered responsible for cytotoxicity (68, 73).

In previous studies, in which TM cells were exposed to 200 μM H_2_O_2_ for 30' (68), or 300 μM H_2_O_2_ for 1 h (74), or to 1 mM H_2_O_2_ for 24 h (71, 72), the typical changes of glaucomatous TM have been observed, including the promotion of cellular senescence (73), rearrangement of cytoskeleton structure (68), and the increase in proinflammatory mediators such as IL-6, IL-8, and endothelial–leukocyte adhesion molecule 1 (ELAM-1) (71).

In our model, on the other hand, TM cells of human origin (HTMC) were exposed to 500 μM H_2_O_2_ for 2 h every day followed by 22 h of recovery, in order to study the effects of oxidative stress administered in chronic/subtoxic manner. Several studies reported that, in the presence of cells, the H_2_O_2_ half-life is very short (~1 h) (75, 76) because it passes rapidly through the cell membranes, and then, it is either detoxified by intracellular enzymes or converted to the above-mentioned reactive hydroxyl radical. Therefore, in our experimental model, 2 h a day of 500 μM H_2_O_2_ were sufficient to exert the cytotoxic/proinflammatory effect on TM cells (6).

## Advanced Human 3D TM Model

In the previous section, we have reported the most recent results of three-dimensional culture models of trabecular meshwork (TM) and the LOC technology. In both cases, it has been demonstrated that the cells cultured in a more similar way to their native-derived tissues provide physiological responses to different stimuli.

Moreover, also in our previous study, we showed the improved suitability of HTMCs (Cell Applications Inc., San Diego, CA, United States) (6, 25) cultured in a 3D model compared to the 2D one, both in basal conditions and after prolonged oxidative stress conditions. In our 3D culture model, we have chosen a degradable scaffold, namely, Corning^®^ Matrigel^®^ Matrix (Corning Life Sciences, Tewksbury, MA, United States) [dx.doi.org/10.17504/protocols.io.574g9qw], which consists of several proteins found in extracellular matrix (ECM) such as laminin, collagen IV, heparin sulfate proteoglycan, and entactin/nidogen (61).

However, the basic requirements for both viability and the functionality of cells, like *in vivo* conditions, are not only cell morphology maintenance but also the continuous supply of nutrients and oxygen, as well as the removal of metabolic waste products (77). Indeed, a more reliable cell and/or tissue micro-environment provides several complex biological responses such as cellular proliferation, migration, differentiation, matrix production, and apoptosis, similar to either the original organ or the tissue in which they arise (78).

Therefore, the development of 3D culture models, which are able to recapitulate some of the critical cell features including cell-to-cell and cell–ECM interactions, are not sufficient to study both the biochemical and biomechanical changes found in a complex disease such as glaucoma (79, 80).

Until today, there have been no standardized advanced *in vitro* models in ophthalmology consisting of both 3D culture models and milli-fluidic techniques for improving the physiological relevance of 3D cultures.

Here we describe the methodology combining a 3D human trabecular meshwork (TM) model and a bioreactor system, in order to overcome the issues related to cell responses under static culture conditions. In fact, the milli-fluidic technique offers precise control over gradients in a continuous manner under milli-metric-size channels (66, 81).

**Figure 1:**
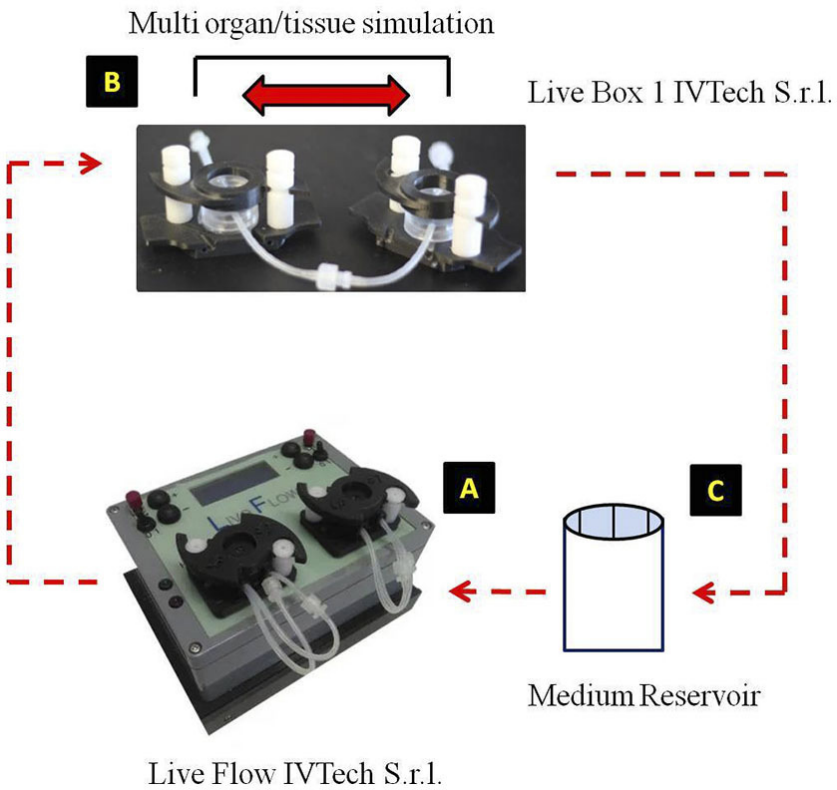
Advanced human 3D model experimental design. From the medium reservoir **(C)**, the medium was pumped by the action of the live flow **(A)**, through the Live Box1 where 3D-trabecular meshwork (TM) cells of human origin (HTMC) were seeded **(B)**, then it returns to the medium reservoir, completing the circuit (Kind permission of IVTech S.r.l.).

In our advanced *in vitro* model (82), the closed-circuit, the 3D-HTMCs received a constant medium supply, consisting of the single-flow bioreactor (Live Box1, IVTechs.r.l., Italy) culture chambers connected to a peristaltic pump (LiveFlow, IVTechs.r.l; Italy) (Figure 1). The medium flow was maintained at a constant rate of 70 μl/min to overcome both diffusional limitations and soft gel degradation on 3D-HTMC. The peristaltic pump takes the culture medium from a 10-ml final volume, so the percentage of turnover rate is 0.7%.

In order to study the effects of oxidative stress (OS) on TM, which is one of the main causes of TM damage, TM cells were treated with daily doses of 2 h of 500 μM H_2_O_2_, and in the remaining time (22 h), they were subjected to recovery under dynamic conditions (40, 76).

The milli-fluidic technology as well as the 3D culture model were capable of mimicking the cell responses found *in vivo* as a result of the increase in outflow resistance. In our model, therefore, it was possible to measure the expression of genes related to a specific cellular activity or function after OS conditions. Indeed, quantitative real-time PCR (qPCR) assays have proved to be a useful tool for assessing the expression of individual genes in order to measure the production of mRNA encoding for both profibrotic and metalloproteinase (MMPs) markers (40, 83–87). Moreover, both the changes in proinflammatory cytokine transcriptions and the NF-kB protein levels have been investigated as markers of activation of inflammatory response following OS stimulation. Finally, the apoptosis protein array was assessed to evaluate also the apoptosis pathway involvement during the experimental time. The increase in inflammatory and profibrotic markers as well as MMPs together with the absence of apoptosis led us to assume that a more efficient adaptive response to OS damage over time may be due to the constant medium supply to cell cultures (40, 71, 73).

**Figure 2:**
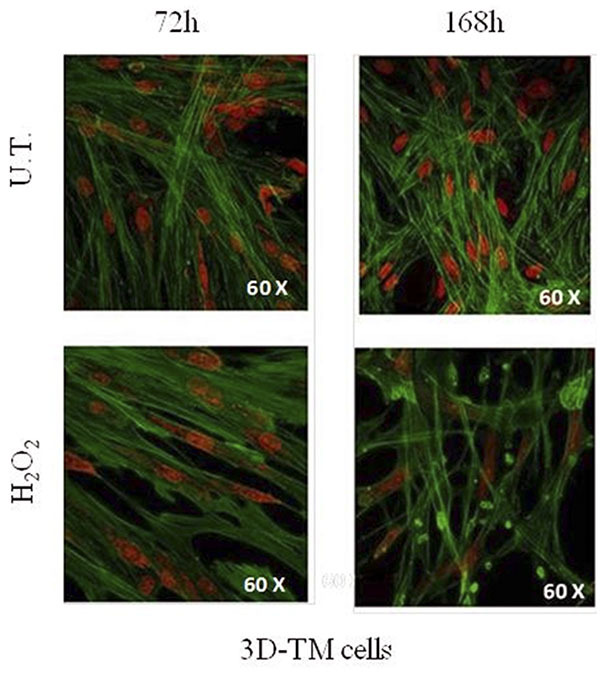
Morphological changes in 3D-TM cells. Confocal analysis of nucleus and cytoskeletal markers were performed on untreated 3D-HTMC (top) and treated 3D-HTMC (bottom) after 72 and 168 h of experimental procedures. Representative images are related to immunoreactivity for To-PRO™ and Phalloidin, as nuclear and cytoskeleton markers, respectively. Merged images showed cytoskeleton plus nucleus (Image was published in Saccà et al. (40) “An Advanced *In Vitro* Model to Assess Glaucoma Onset”).

In addition, the morphological comparison between 3D-HTMCs cultured under static conditions and those cultured under a dynamic one (Figure 2) showed, at the longest experimental time tested (168 h), a recovery of cytoskeleton integrity only in the advanced *in vitro* model, as confirmed also by the cell viability assay.

The perfusion of culture media through a 3D-culture structure using a pump system provide the proper nutrient circulation, metabolic waste expulsion, and homogeneity of the physical and chemical factors, which, in turn, allow for the study of early OS-derived molecular change without inducing the premature apoptosis as it happens under static culture condition. Therefore, bioreactor-based cell culture models are appropriate to study the stimuli-derived biomolecule cell productions to better understand the early alterations, which occur in glaucoma first steps (25).

Indeed, this 3D-TM milli-fluidic model represents a useful tool for providing a physiological cellular environment under controlled experimental conditions.

In addition, another way to think of this technology is either to combine the different modules/chambers in series for mimicking the tissue crosstalk between different tissues or equip it with a device to induce an increase in basal medium flow pressure. In these ways, this *in vitro* model would make it possible to analyze step-by-step the stages of cell damage, which underlie glaucoma and its adverse outcomes.

## Conclusions and Future Prospects

During the past three decades, a large number of studies have demonstrated that the increase in ROS rate, from endogenous and exogenous sources, can cause an unbalance in cell redox state, which leads to cellular damage (88, 89). Indeed, it is now known that the direct effects of the oxidative stress (OS) injury underlying the POAG onset seem to be linked in particular with TM damage, which is responsible for the increase in IOP in glaucomatous eyes (26–29). However, given the crucial role of TM in conventional outflow pathway regulation, it has also been hypothesized that glaucomatous TM could be involved in RGC death through the release of molecular signals harmful for RGC. Thus, molecular modifications borne by damaged TM tissue such as alterations in gene and protein products may affect the RGC viability (25).

In this review, we emphasize the importance of studying the first molecular changes in TM after oxidative stress by using an advanced human 3D-TM model. The novel therapeutic approaches are taking into account TM as a possible pharmacological target (90, 91). Therefore, the possibility of using an advanced *in vitro* model could represent an important tool for analyzing the cellular responses of a single-cell population, which in this case is represented by TM.

Currently, the lack of experimental standardization for glaucoma study neither for *in vitro* nor for animal models (e.g., design, sample size, analytical techniques, statistics, reproducibility, lack of specific species, and so on) has partly compromised the proper identification of glaucoma biomarkers. Previous *in vitro* studies on TM have undoubtedly helped to understand the molecular changes in TM following OS treatment; however, most of the clinical trial results based on small animal models (i.e., rodents) revealed their translational failure mainly due to the different degree of similarity between human disease and the animal model used (21, 26, 92–94).

The TM is a porous tissue, and its structure is made up of connective tissue beams and sheets or lamellae covered by TM cells. Since the TM function is to filter AH, the 3D structure is a very important parameter to evaluate. In particular, in the 3D TM model *in vitro* proposed here, the TM cells embedded in a natural hydrogel seem to be more sensitive to OS, reflecting the OS-derived TM degeneration in a more realistic way (40). Furthermore, the cross-talk between cells, favored by 3D morphology, allows a more physiological cell-to-cell interaction (i.e., cytokines secretion) such as to promote both cell survival and cell proliferation even after harmful stimuli (e.g., oxidative stress) (40, 79). Moreover, our 3D TM *in vitro* model maintains power law metabolic scaling in cultures proving the physiological relevance for such a down-scaled *in vitro* system (95). In addition, the TM is an essential tissue, which together with a complex organ system, plays a pivotal role in modulating AH outflow (96, 97). In physiological conditions, the balance between aqueous humor inflow and outflow rate regulates the IOP in order to maintain the shape and related refractive properties of the eye. In relation to this, our innovative platform has been improved by adding an auxiliary device (Live Pa, IVTech S.r.l) to the millifluidic circuit in order to study the effects of increased flow pressure on TM cells (Figure 1) (98, 99).

Moreover, thanks to the features of this advanced *in vitro* model, which allows multiorgan approach through the communication between different tissues, it can be used to study the involvement of different cell types in glaucoma (e.g., neuron-like cells). In this way, it will be possible to evaluate how the TM damage, due to oxidative stress and/or increased pressure, influenced neuron homeostasis. In addition, such platform will allow to check the effectiveness of therapeutic compounds in counteracting the oxidative and/or pressure damage during the glaucoma evolution.

## Author Contributions

All authors listed have made a substantial, direct and intellectual contribution to the work, and approved it for publication.

## Conflict of Interest

The authors declare that the research was conducted in the absence of any commercial or financial relationships that could be construed as a potential conflict of interest.
